# A decade of deprioritisation? Ethnicity and health in the 10-year NHS plan

**DOI:** 10.1177/01410768251366877

**Published:** 2025-08-11

**Authors:** RA Powell, K Bhui, N Singh, M Rao, G Sathyamoorthy

**Affiliations:** 1 Ethnicity and Health Unit, Department of Primary Care and Public Health, 4615 School of Public Health, Imperial College London, London W12 0BZ, UK; 2 Department of Psychiatry, Warneford Hospital, Medical Sciences Division, University of Oxford, Oxford, UK; 3 Chelsea and Westminster Foundation Trust, London, UK

Speaking in Blackpool in advance of the Government’s much-anticipated *10-Year Health Plan* launch, Health and Social Care Secretary Wes Streeting cited the ‘inverse care law’: ‘those in greatest need often receive the worst-quality healthcare’.^1^ He promised the Plan would tackle these inequalities through fundamental reforms.

The Plan’s subsequent publication was heralded by the Government as a fundamental rewiring for the National Health Service (NHS)’s future. Central to the document are the three strategic shifts in service provision – hospital to community, sickness to prevention and analogue to digital – and the mantra that everyone deserves the same quality treatment.^2^

The Plan’s release provoked mixed reactions. A warm welcome by Advancing Quality Alliance,^3^ which celebrated ‘a clear and ambitious direction for the future’, contrasted with a sober reflection on the Plan’s lack of a unifying theory of change to implement its ideas,^4^ and an outright condemnation by the *Health Service Journal*, which hailed it ‘a mess’.^5^

Responses focused on the Plan’s equality, diversity and inclusion (EDI) elements have been relatively muted. The NHS Race and Health Observatory (NHS RHO) has broadly embraced it, highlighting governmental commitment to: NHS recruitment from served communities, with recruitment data disaggregated ethnically; working to create staff standards and tackle workplace racism; leveraging the NHS App to provide personalised care to ethnic minority patients; the possibility of using patient datasets with improved recording of ethnicity; and widespread NHS adoption of artificial intelligence (AI), creating opportunities to address racial biases that are structurally embedded in machine learning systems and large language models.^6^

Few would disagree with the Plan’s direction of travel, even if the words ethnic/ethnicity are only mentioned six times in its 168 pages. But we propose three areas that need to be considered in future government pronouncements: health recipients, health research and health providers.

## Health recipients

Ethnicity-based health inequalities that persist across the life course are widespread in the UK. These inequalities are systemic and syndemic in nature. Not only the consequence of individual behaviours or isolated diseases, they are also interlinked outcomes of biological vulnerability, intersectional-based social disadvantage and systemic and historical racism.^7^ Key among these factors are racism and discrimination, poor housing and environmental conditions, precarious employment and low income, and limited access to effective and culturally appropriate healthcare. These entangled, mutually constitutive and interacting elements can result in poorer outcomes, partially mediated by delays in seeking healthcare and mistrust of health authorities. In this context, ‘non-compliance’ and being ‘hard to reach’ are rational responses to cumulative societal exclusion and potentially coercive or harmful interactions.

By bundling the complex needs of ethnic minority communities from economically deprived areas with other geographically-based disadvantaged groups (i.e. rural communities, coastal towns and deprived inner cities), their intersectional disadvantages can be erased from the data and neglected. Moreover, addressing such deeply rooted challenges requires more than traditional ‘health provision’ and NHS demand management. It needs cross-departmental planning, working across government to tackle the issues’ holistic nature. A genuine shift towards equity must move beyond narrowly-focused service delivery paradigms and address the eco-social, cultural-structural, and syndemic determinants that drive health inequalities.

## Health research

The Plan’s support for new research investigators in under-represented areas, and the use of ‘research inclusion’ as a new condition for National Institute for Health and Social Care Research funding, is welcome. However, an opportunity has been missed to emphasise the critical role, especially among marginalised ethnic minority communities, of engaging with health research, and operationalising what research inclusion means for different populations and contexts.

High quality healthcare provision is premised upon clinical expertise, patient values and the conscientious, explicit, judicious use of modern, best evidence.^8^ This evidence must be comprehensive and inclusive, culturally embracing and relevant to address the needs, priorities and preferences of the populations it seeks to serve. If not, insufficient representation will result in neglect and disproportionately poor health outcomes, and people missing out on the important benefits of research. There will also be limitations to the applicability and generalisability of research findings. Research often demonstrates a disproportionate lack of participation in health research among people from marginalised communities,^9^ a fact exemplified during the development of a COVID-19 vaccine.^10^

National Health Service England (NHSE) has been at the forefront of this agenda, supporting research engagement with communities, based on a ‘working with, not on’ axiom. For example, in north-west London, the Research Engagement Network programme has worked closely with local communities, voluntary, community and social enterprise organisations and the Integrated Care Board to break down myths around research,^11^ and encourage participation in health research. Creative, participatory, adapted methodologies must sit alongside and within existing trials and data platforms, which continue to under recruit the most deprived marginalised groups with the poorest health indices. NHSE’s work in this area should be brought under the umbrella of the reconstructed Department of Health and Social Care.

## Health providers

The Plan scales back the *2023 NHS Long-Term Workforce Plan*,^12^ with international medical graduate (IMG) – that is, those who are qualified overseas – recruitment deprioritised. A focus on growing training places and recruitment from within the UK aims to reduce the NHS’s dependence on overseas doctors. It is anticipated that by 2035 less than 10% – rather than the existing 34% – of new hires will be from overseas.^13^

While there is a widely recognised moral imperative to reduce the number of health staff sourced globally – especially from ‘red list’ countries, where health systems are being denuded of workers – IMGs will still comprise a significant percentage of the NHS workforce for the foreseeable future. As Streeting himself noted at the recent NHS Expo Conference: ‘If they [migrant workers] all left tomorrow, the NHS would collapse’.^14^ Transitioning IMGs into their new NHS roles, as well as UK cultural life, therefore remains critical, helping IMGs to provide quality patient care.

In 2022, a national induction programme was launched that guided NHS Trusts in how this induction could be effectively introduced.^15^ However, its uptake has been patchy at best. While the Plan makes implicit reference to IMGs – describing the NHS as ‘proud of its international workforce’ (see Department of Health and Social Care^2^, p. 102), no mention is made of their induction and how Trusts can be obligated to adopt the national guidance. This oversight is critical. A failure to induct IMGs properly not only affects the wellbeing and retention of these vital staff members but can also directly impact the quality and cultural competence of patient care.

There is much that is potentially transformative about the *10-Year Health Plan* and Wes Streeting’s promise to tackle existing entrenched inequalities across the country. Our concerns regarding ethnicity and health recipients, health research and health providers are more widely premised on a concern that the EDI agenda is slipping down political priorities. If it is, and ethnicity is being deprioritised, those in greatest need – that include those from ethnic communities – will continue to receive inequitable healthcare.

## Declarations

### Guarantor

GS.

### Provenance

Not commissioned; editorial review.
